# Introduction of Air in the Veins

**Published:** 1843-06

**Authors:** 


*Introduction of Air in the Veins.*—The "Annales de la Chirurgie Française et Etrangère" contain a communication, by Marchal de Calvi, on the introduction of air into the veins, in the course of which he offers a new explanation of the cause of death produced thereby. The opinion that it depends on the distension of the heart, by which its contractions are prevented, is, he admits, supported by the experiments of Nysten with the different gases, but he says it does not explain those cases where the death is so frightfully sudden, as if the patient had been struck by lightning, and which resembles the immediate effect of a poisonous dose of prussic acid. In these cases there must be, he thinks, some toxic agent, and then arises the question, what is that agent? Hitherto to this there has not been offered any reply, but Marchal thinks he has found the answer, by discovering the presence of carbonic acid gas in the heart, which he is of opinion is disengaged every time air is introduced into it through the venous system. In this he is supported by the appearance of the blood in the pulmonary cavities of the heart, which, instead of being black, is red, it having been decarbonised by the contact with the atmospheric air, the oxygen of which has combined with its carbon, and formed the carbonic acid gas, to the action of which Marchal refers the death of the patient; and again, if, in performing experiments on this subject, the operator breathe through the tube into the vein, instead of injecting the purer air around him, the fatal effect will be much more rapidly induced, there being carbonic acid gas already generated in the injected fluid. The experiments of Nysten, however, with carbonic acid gas and oxide of carbon do not support this hypothesis.—*Med. Examiner,* from *Provincial Medical Journal.*

